# Uncovering the special microbiota associated with occurrence and progression of gastric cancer by using RNA-sequencing

**DOI:** 10.1038/s41598-023-32809-9

**Published:** 2023-04-07

**Authors:** Bin Ai, Yue Mei, Dong Liang, Tengjiao Wang, Hui Cai, Dong Yu

**Affiliations:** 1 Department of Precision Medicine, Translational Medicine Research Center, Naval Medical University, Shanghai, China; 2 Shanghai Key Laboratory of Cell Engineering, Shanghai, China; 3 Department of Gastrointestinal Surgery, Changhai Hospital, Shanghai, China

**Keywords:** Cancer, Microbiology

## Abstract

Gastric cancer (GC) has been identified as the third deadly cancer in the world. Accumulating researches suggest a potential role of microorganisms in tumorigenesis. However, the composition of microbiota in GC tissues is not clear and it changes throughout the different stages of GC remain mostly elusive. Our study integrated RNA-Seq data of 727 samples derived from gastric tissues across four datasets and revealed its microbial composition. In order to remove the false positive results, core taxa were defined and characterized. Based on it, we analyzed the influence of biological factors on its composition. The pan-microbiome of gastric tissues was estimated to be over than 1400 genera. Seventeen core genera were identified. Among them, *Helicobacter*, *Lysobacter* were significantly enriched in normal tissues, while *Pseudomonas* was enriched in tumor tissues. Interestingly, *Acinetobacter*, *Pasteurella*, *Streptomyces*, *Chlamydia*, and *Lysobacter*, showed a significant increase trend during tumor development and formed strong intra/inter-correlations among them or with other genera. Furthermore, we found that tumor stage played an important role in altering the microbial composition of GC tissues. This study provides support for the in-depth study of tumor microbiome, and the specific microbiome excavated provides a possibility for the subsequent identification of potential biomarkers for GC.

## Introduction

Gastric cancer (GC) is a malignancy of the gastrointestinal tract that has been identified as the fifth most common cancer and the third deadly cancer in the world^1^. The rate of early diagnosis is low and most patients are often diagnosed at a late stage, directly leading to miss the best clinical treatment period^2^. The prognosis of advanced gastric cancer is undesirable and the 5-year survival rate after surgery is less than 30%^3^. Although the incidence and mortality of gastric cancer have decreased in recent years, gastric cancer maintains a high fatality rate of 75% in most parts of the world^4^. In recent decades, various studies have analyzed the mechanism of its carcinogenesis through sequencing technology, especially to explore the relationship between specific microbes and the development of gastric cancer^5,6^. However, there is still a lack of effective molecular markers for early screening and diagnosis of gastric cancer, and new methods are urgently needed to mine new biomarkers.

Recently, more studies have shown that microorganisms play a potential role in tumorigenesis and cancer therapies. With the application of high throughput sequencing technology in microbiology, it was found that the stomach, once considered as an organ where microorganisms could not thrive, was colonized by robust microbiota^7^. Aviles-Jimenez suggested that as patients progressed from superficial gastritis to intestinal-type gastric cancer (GC), the bacterial diversity of tissues decreased at the genus level^8^. In addition, several studies found that the microbial diversity of gastric cancer patients was significantly lower than that of chronic gastritis patients^9,10^. Ferreira found that compared with patients with chronic gastritis, intestinal symbiotic bacteria such as *Achromobacter, Lactobacillus, Citrobacter, Clostridium and Rhodococcus* were significantly more abundant in the bacterial community of gastric cancer patients^10^. Chen demonstrated that *Helicobacter pylori* abundance was significantly decreased in gastric cancer compared with non-tumor tissue, while some oral bacteria such as *Peptostreptococcus*, *Streptococcus*, and *Fusobacterium* were enriched in gastric cancer tissue^11^. Notably, a set of studies have already proved that *Helicobacter pylori* colonizes the stomach and its infection is a strong risk factor for gastric cancer^12,13^. In conclusion, gastric microbiome change during the progression from normal and healthy gastric mucosa to gastric cancer and specific microbes play a potential role in this process.

However, until recent years, the relationship between the non-H.pylori microbiota and gastric cancer is still inconclusive and unexplored. For example, whether the microbiome of gastric cancer patients increases or decreases compared to healthy individuals is uncertain. Whether the increase or decrease of certain microorganisms is related to the occurrence and development of gastric cancer? Whether other risk factors for gastric cancer (including advanced age and male gender) can affect the microbiota of gastric cancer tissue? More investigation is needed to help to better explore the role of microorganisms in the progression of gastric cancer.

In the era of high-throughput sequencing, 16S rRNA sequencing is frequently utilized in microbiome studies^14^. In addition, omics data mining provides a novel approach to study microbial profile, including RNA-seq data, WGS data and Metagenomic sequencing data, which can calculate and analyze by microbial classification engines such as Kraken2^15^, Centrifuge^16^, DIAMOND^17^ and MetaPhlAn2^18^ to mine the microbial information. These new techniques provides more information about the composition of the bacterial community than traditional cloning and sequencing methods. Among them, Kraken2 has been found to be very reliable in microbial identification in a large number of studies, especially in microbiome studies based on RNA sequencing data^19^. Poore has been provided a valuable and feasible approach to discovering unique microbial information by analyzing genome-wide and transcriptomic data from blood and tissues of cancer patients^20^. Therefore, these techniques can help us to better characterize the GC tissue microbiota.

In this study, four transcriptome datasets from GC tissues, including 727 samples, were used. Microbial reads of each sample were obtained by kraken2 algorithm and then integrated to reveal the microbial profile of GC tissues. In addition, we explored the influence of biological factors on tumor tissue microbiome. Furthermore, the core microorganisms related to the occurrence and development of gastric cancer were revealed. The results not only provide support for subsequent studies related to the tumor microbiome, but also provide the possibility to identify new biomarkers for gastric cancer.

## Methods

### Data selection and processing

We performed a search of the dataset in NCBI Gene Expression Omnibus (GEO database) using the key words “gastric cancer” and “expression profiling by high throughput sequencing”. Notably, datasets without detailed biological information or with a small sample size were excluded. Finally, four transcriptome sequencing datasets, SRP172499, SRP337610, SRP326473 and SRP330001, were included in this study. The raw data from all datasets were downloaded in the sequence read archive (SRA) format and converted into FASTQ format using SRA Toolkit. The raw fastq files were then evaluated, trimmed and filtered for quality control using software FastQC and Trim Galore, respectively. To remove human reads from sequenced reads, quality-controlled sequenced reads were mapped to the human reference databases.

### Microbial detection and visualization

For microbial identification, the sequences were mapped to microbial reference database using Kraken2, a taxonomic classification system that uses exact *k*-mer matches to achieve high accuracy and rapid classification. The microbial reference database contains nearly all known fungal, bacterial, archaeal and viral genomes. The Kraken2 outputs were integrated in R-software (v.4.2.1). The results of Kraken2 analysis were visualized using Krona software, which displays hierarchical aspects of the taxonomy and statistics about each taxon in multi-layered pie charts. The following analyses were conducted at the genus level of assigned taxa.

### Microbial profile analysis

The output file was further analyzed using R-software packages, phyloseq (v.1.41.0) and microbiome (v.1.19.0) and visualized using R packages VennDiagram (v.1.7.3) and ggplot2 (v.3.3.6). Core microbiota were defined as that identified at a minimum positive detection rate, present in the majority of the population. Identification of core microbiota was performed under 0.2% positivity detection and 20% prevalence^21^. Calculation of pan microbiome according to research methods to test the openness/closeness of the microbiome^22^. Diversity measurements of alpha diversity and beta diversity were performed in each dataset using the vegan R-package (v.2.6.2) according to its different biological characteristics. The R program package “tidyverse” (v.1.3.1) and “rstatix” (v.0.7.0) were used to render STAMP result graph. Correlation analysis was performed using R program packages “igraph” (v.1.3.2) and “psych” (v.2.2.5) and considered correlation coefficients greater or < 0.6 and − 0.6 respectively and *P* < 0 0.05. Visualization of the co-occurrence network was achieved using Gephi version 0.9.2.

### Statistical analysis

Significantly different microbes among groups were screened out by the Wilcox test (applied for 2 groups) and two-way ANOVA (applied for more than 2 groups). PERMANOVAR was used to quantify multivariate community-level differences in microbial composition among groups. For correlation analyses, Spearman's rank correlation test was used. STAMP algorithm was used to identify microorganism differences between groups (Bonferroni correction method). *P* < 0.05 was considered to be significant.

## Results

### Dataset characteristics

Four gastric cancer (GC) datasets with RNA sequencing data were selected for this study (SRP172499, SRP337610, SRP326473, SRP330001), which contained 160, 461, 76, and 30 samples, respectively. The meta information of each dataset was collected by literature search, as shown in Table 1. Notably, each dataset has its special focus beyond other common factors: tissue type for SRP172499, tissue source for SRP330001, tumor stage for SRP337610 and age for SRP326473. Additionally, all the samples we analyzed were from Asians.

**Table 1:** Characteristics of the samples in each dataset.

Study	SRP172499	SRP337610	SRP326473	SRP330001
DataSubmittedLab	Korea University	Harbin Medical University Cancer Hospital	Harbin Medical University Cancer Hospital	Peking university
Sequencing platform	HiSeq	HiSeq	HiSeq	BgiSeq
Tissue type				
Normal	80	230	38	–
Tumor	80	231	38	30
Tissue source				
Fresh	–	–	–	30
Flash frozen	160	461	76	
Gender				
Male	–	295	48	–
Female	–	166	28	–
Age				
≤ 50	160	–	26	–
> 50	–	–	50	–
Tumor stage				
IA	–	18	–	–
IB	–	18	–	–
IIA	–	25	–	–
IIB	–	24	–	–
IIIA	–	47	–	–
IIIB	–	50	21	–
IIIC	–	32	17	–
IV	–	17	–	–
Histology				
Intestinal	6	–	–	–
Diffuse	148	–	–	–
Mixed	4	–	–	–
MSI				
MSS/MSI-L	152	–	–	–
MSI-H	8	–	–	–

“–” means NA, which indicates that the dataset does not possess the characteristic.

### Microorganisms are present in the microenvironment of gastric tissues

**Figure 1:**
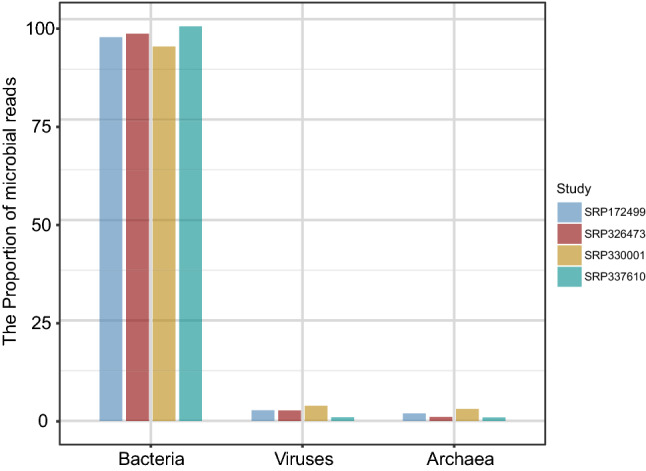
The proportion (%) of bacteria, viruses, and archaea in identified microbial reads in each dataset.

The microbial profiles were detected from the four selected gastric tissues datasets using Kraken2 algorithm. Microbial reads were found in all the samples, which were further assigned to the taxa of bacteria, archaea and viruses. The bacterial reads in each dataset took the highest proportion (> 90%) (Fig. 1). And less than 4% of the reads were detected as virus in each dataset, while the archaea reads take a negligible proportion (Fig. 1). The microbial composition of each dataset was shown in Krona plot (Supplementary Figs. S1 and S2). There results suggested that microorganisms, especially bacteria, did exist and take a dominant role in the microenvironment of gastric tissues.

### Microorganisms present in gastric tissues are highly conservative and stable

**Figure 2:**
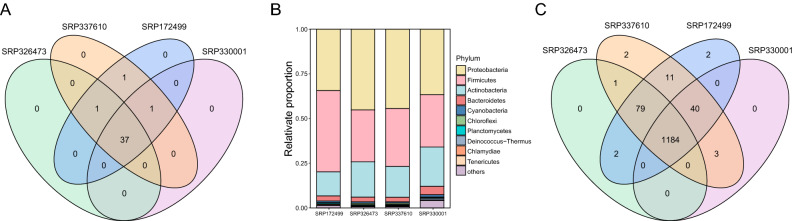
The overlap of the microbial profiles across the four datasets at the phylum and at the genus level, and the relative abundances of the top 10 phylum among the four datasets. (**A**) Venn diagram showing the overlap of the microbial profiles across the four datasets at the phylum level. (**B**) The relative abundances of the top 10 phylum. (**C**) Overlap of the microbial profiles across the four datasets at the at the genus level by Venn plots.

Next, we further investigated the microbial composition at different taxonomical level across the datasets. At the phylum level, a total of 40 phyla were identified, of which 37 were shared by the four datasets (Fig. 2A). The relative abundances of the top10 phylum in each dataset were shown in Fig. 2B. *Proteobacteria*, *Firmicute*, *Actinobacteria*, and *Bacteroidetes* were the most prevalent and abundant phylum across the four datasets (Fig. 2B). The distribution of these phyla across the samples were showed in Supplementary Fig. S3. At the genus level, a total of 1324 unique genus were identified across all samples, of which the four datasets contained 1266, 1320, 1318 and 1227 taxa respectively (Fig. 2C). From these, more than 89.7% (1184/1320) of the microbial composition in each dataset were shared, suggesting that the microbial composition of the gastric tissue microenvironment was highly conservative and stable.

**Figure 3:**
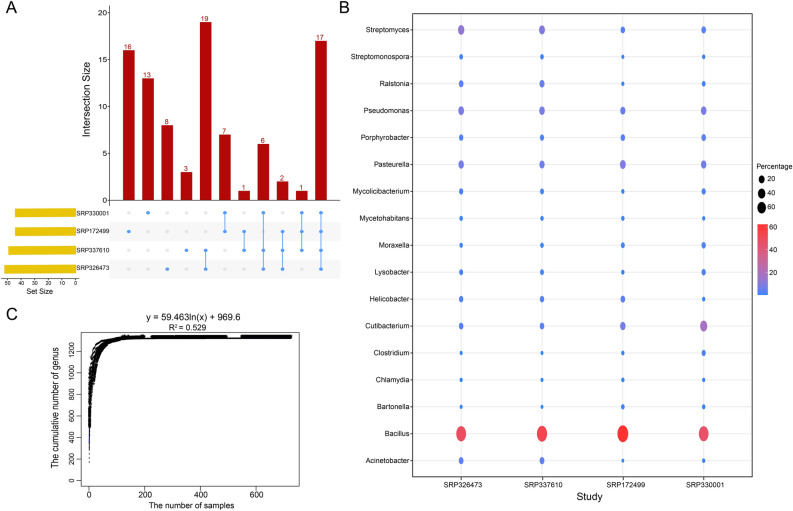
Core microbial profiles and estimation of the size of pan-microbiome in GC tissue. (**A**) UpSet plot of the core microbial profiles across the four datasets at the genus level. The horizontal axis represents the number of core genus in each dataset. The vertical axis represents the number of core genus for one or several datasets. (**B**) The relative abundances of the 17 genera in 4 datasets. (**C**) The accumulation curve of GC tissues.

In order to remove the false positive results, core taxa were defined and characterized. There were 49, 44, 44, and 52 genera identified as core microbiota in dataset SRP337610, SRP172499, SRP330001 and SRP326473 respectively (Fig. 3A). Among them, 17 genera: *Acinetobacter, Bartonella, Helicobacter, Moraxella, Mycetohabitans, Pasteurella, Porphyrobacter, Ralstonia, Bacillus*, *Pseudomonas*, *Streptomyces*, *Cutibacterium*, *Lysobacter, Mycolicibacterium*, *Clostridium*, *Streptomonospora*, *Chlamydia*, were shared. The distribution of them in each dataset was shown in Fig. 3B. Among them, *Bacillus* genus was reported as a relatively common microorganism with higher abundance in the composition of gastric microbiota^23,24^. *Helicobacter pylori* is a well-acknowledged carcinogen bacterial and its infection significantly increases the risk of gastric cancer^25^.

In order to generally estimate the microbial abundance of gastric tissues, the concept of “Pan-genome” was referred. We modelled accumulation curves from the genus across our 727 samples to check the openness/closeness of the pan-genome (Fig. 3C). As expected, the number of the genera increased continuously as the number of considered samples increased, but the pan microbial profile in gastric tissues appears not to have been reached. Therefore, there are more microbes in gastric tissues than previous known, which should be paid more attention in future research studies.

### Influence of biological factors on microbial diversity and composition in GC tissue

Two indicators, alpha diversity and beta diversity, can quantify the diversity of a particular microbial community. Then the influence of the biological factors on the microbial diversity and composition in each dataset were explored. In addition, differential analysis was also conducted to identify the significantly differential genus related with these factors.

### Tissue type

**Figure 4:**
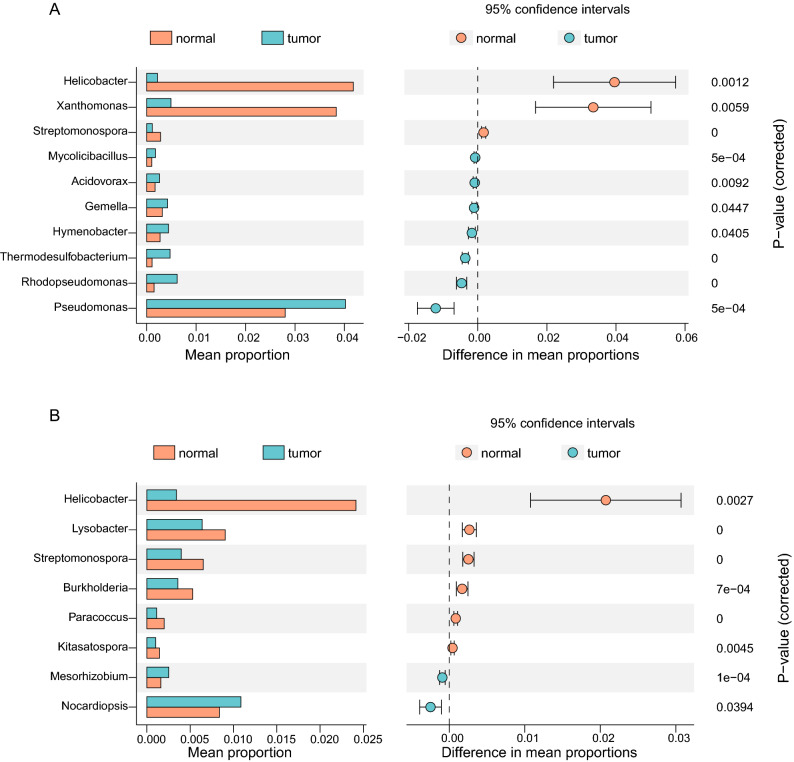
The different taxa were evaluated between the normal and tumor tissue based on Wilcox test in the dataset SRP172499 (**A**) and in the dataset SRP337610 (**B**).

There were tumor and adjacent normal samples in the datasets SRP326473, SRP172499 and SRP337610. However, no significant differences were detected in alpha diversity using Shannon index within the three datasets (Supplementary Fig. S4A–C). We found the tumor group has lower inter-divergence values than the normal group and only the dataset SRP172499 had significant difference between the two groups (Supplementary Fig. S4D–F). The tissue type (normal *vs.* tumor) had no significant effect on the overall microbial composition in datasets SRP326473 (PERMANOVA test, *P* = 0.41), but had significant effect in the other two datasets (PERMANOVA test, *P* < 0.001 *** for SRP172499 and SRP337610). The small sample size of SRP326473 dataset might make it statistically insufficient to detect potential differences in microbiome between groups. To evaluate whether there were differences in taxa that could explain the variation in diversity, the composition of the taxa between normal and tumor samples were compared by STAMP (Fig. 4). In the dataset SRP172499, *Helicobacter*, *Xanthomonas* and *Streptomonospora* were enriched in the normal group, while *Mycolicibacillus*, *Acidovorax*, *Gemella*, *Hymenobacter*, *Thermodesulfobacterium*, *Rhodopseudomonas* and *Pseudomonas* were enriched in the tumor group (Fig. 4A). However, in the dataset SRP337610, *Nocardiopsis* and *Mesorhizobium* were enriched in the tumor group and six genera, *Helicobacter*, *Lysobacter*, *Burkholderia*, *Paracoccus*, *Streptomonospora* and *Kitasatospora* were enriched in the normal group (Fig. 4B). In both datasets, *Helicobacter* was significantly enriched in normal tissues, which is consistent with previous findings^26^.

### Gender

The datasets SRP337610 and SRP326473 had the gender phenotype for each sample. The gender group (female *vs*. male) had no significant effect on the overall microbiota composition in either of the datasets (PERMANOVA test, for SRP326473, *P* = 0.71; for SRP337610,* P* = 0.36). The similar results were observed in Shannon diversity between the female and male samples (Supplementary Fig. S5A, B). Although there was no significant difference in the beta diversity of the gender group in the SRP326473 dataset, the beta diversity of the female group was significantly higher than that of the male group in the SRP337610 dataset, which had a larger sample size (Supplementary Fig. S5D, E). The results showed that the overall community structure of gastric tumor tissue was similar in different genders, but the heterogeneity of gastric tumor tissue microorganisms might be higher in female patients.

### Age

Age is a well-known risk factor for gastric cancer and the incidence rate of GC increases gradually with age. The dataset SRP326473 contained the phenotype of age for each sample. In order to explore the effect of age on microbial composition, the samples were divided into two groups by age 50: Old (> 50 year old), and Young (≤ 50 year old)^27^. PERMANOVA analysis showed that the age group (Old *vs*. Young) also had no significant effect on the microbiota composition (*P* = 0.27). However, the definition of young age or old age GC remains controversial, so we also set different thresholds ranging from 40 to 65 were set to re-define the age groups^28^. There were still no significant differences between the Old and Young group, suggesting a stable composition after tumorigenesis. As for diversity, there were no differences in Shannon index between the Old and Young groups (Wilcox test, *P* = 0.50) (Supplementary Fig. S5C), but there were significant differences in divergences within the two groups (Wilcox test, *P* = 0.014) (Supplementary Fig. S5F). Beta diversity was significantly increased in the old group, suggesting that the patients with older age may have a more heterogeneous tumor microbiome in the GC tissue.

### Tumor stage

**Figure 5:**
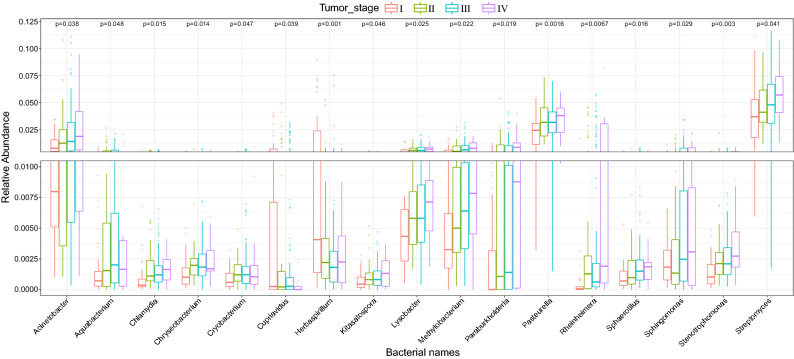
Seventeen differentially abundant bacterial genus were identified among the four tumor stages (*P* < 0 .05, ANOVA).

The datasets SRP337610 and SRP326473 contained the tumor stage for each sample. The samples from dataset SRP326473 had tumor stage IIIB and IIIC. There were detailed tumor stages in the SRP337610 dataset, including IA, IB, IIA, IIB, IIIA, IIIB, IIIC and IV. Neither the microbial diversity nor composition between IIIB and IIIC were significantly different in the dataset SRP326473 (Supplementary Fig. S6A,B), suggesting that the microbial composition might be more stable in high-level malignant tumor tissues. To further explore the changes in microbial composition at different tumor stages, the present study merged IA, IB into stage I, IIA, IIB into stage II, and IIIA, IIIB, IIIC into stage III. The microbial diversity was not significantly different among the four cancer stages, but the beta diversity has significantly different (Supplementary Fig. S6C,D). We found that as tumor development progressed, the tissue microbial divergences showed a significant decrease. PERMANOVA analysis showed that there were significant differences among the microbial composition of I, II, III and IV samples (*P* = 0.011). 17 genera (*Cryobacterium*, *Kitasatospora*, *Streptomyces*, *Chryseobacterium*, *Chlamydia*, *Methylobacterium*, *Sphingomonas*, *Cupriavidus*, *Paraburkholderia*, *Herbaspirillum*, *Aquabacterium*, *Sphaerotilus*, *Rheinheimera*, *Pasteurella*, *Acinetobacter*, *Lysobacter* and *Stenotrophomonas*) were identified to be significantly different in abundance across the four groups and tended to increase during tumor progression (Fig. 5). In summary, the microbial composition of GC tissue changed significantly during the development and progression of tumors.

### Correlation network analysis

**Figure 6:**
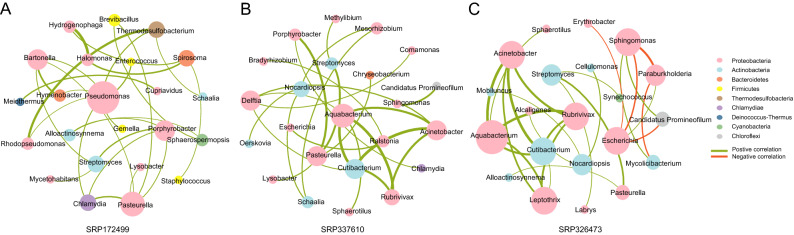
Correlation strengths of the core genera in different datasets. Correlation network of the core genera in the dataset SRP172499 (**A**), SRP337610 (**B**) and SRP326473 (**C**). The green line indicates a positive correlation (r > 0.6), and the red line indicates a negative correlation (r < − 0.6). Each circle represents a core bacterial genus, the color of the circle represents the phylum to which it belongs and the size of the circle is proportional to its relative abundance.

To further understand the potential interaction among core bacterial genera, we performed co-occurrence network analysis in the three datasets. Significant correlations were found in SRP172499 (27 genera pairs), SRP337610 (24 genera pairs ) and SRP326473 (26 genera pairs ) (*P* < 0.05, r > 0.6 or r < −0.6, Fig. 6). The correlation networks formed different bacterial clusters in the three datasets. We could see that the bacterial community is mainly composed of the genera of *Proteobacteria* and *Actinobacteria.* There were five genera pairs appearing negative correlations. Among them, strong negative correlation was formed between *Sphingomonas* and *Parburkholderia. Streptomyces*, *Pasteurella* formed various bacterial clusters in all three datasets and positive correlations were found between them. In addition, *Pasteurella* also had some co-occurrence interactions with *Lysobacter, Porphyrobacter.* Most of the strong positive correlations occurred in the genera of *Proteobacteria*, such as among *Acinetobacter, Ralstonia*, *Delftia*, *Escherichia*, *Aquabacterium*, and *Rubrivivax.* Interestingly, *Cutibacterium* had a strong positive correlation with the genera of *Proteobacteria* (*Aquabacterium*, *Rubrivivax*, *Acinetobacter*). These microorganisms might play a key role in the network.

### Validating microbial signatures in GC tissue

**Figure 7:**
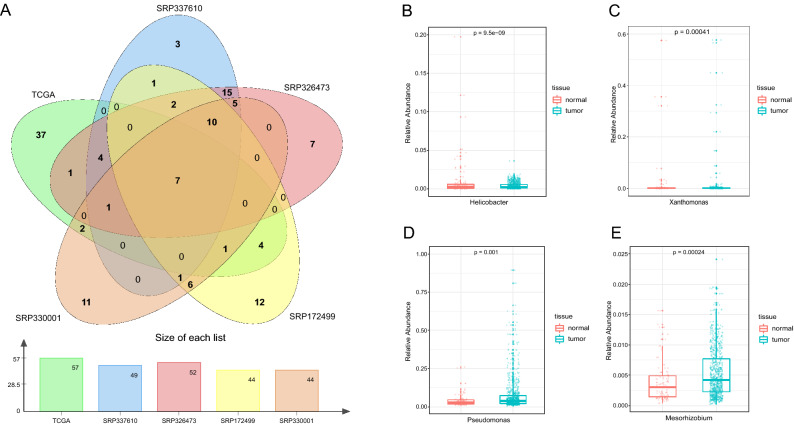
Validation of core genera and differential bacterial genera of the TCGA dataset. (**A**) Venn plot of the core microbial profiles across the datasets at the genus level. Differences in relative abundance of *Helicobacter* (**B**), *Xanthomonas* (**C**), *Pseudomonas* (**D**) and *Mesorhizobium* (**E**) between tumor and normal tissue.

The computational results were validated using TCGA dataset^20^. We also screened the core genera in the TCGA dataset and examined gastric tissue on basis of several significantly different microorganisms. 57 genera were identified as core microbiota in TCGA cohort, of which 7 core microbiota (*Acinetobacter, Bacillus, Chlamydia, Clostridium, Helicobacter, Pseudomonas,* and *Streptomyces*) were also detected in our dataset (Fig. 7A). In the core genera of TCGA, *Helicobacter* was found to be differentially abundant between tumor and normal tissue with higher relative abundance in normal tissue than tumor tissue in TCGA cohort (Fig. 7B)*.* Similarly*, Xanthomonas* was also significantly enriched in normal tissue (Fig. 7C). In addition, *Pseudomonas* and *Mesorhizobium* were found significantly higher prevalence in tumor tissue than in normal tissue (Fig. 7D,E), in which *Pseudomonas* had been confirmed to have certain anti-tumor activity^29^. These consistent results indicated the reliability and validity of our analysis.

## Discussion

At present, more researches focus on the role of microorganisms in the occurrence and development of gastric cancer. However, apart from *Helicobacter pylori*, a definite role for the other microbiota in the development of gastric carcinogenesis has not yet been established. In this study, we integrated four datasets to demonstrate microbial landscape of gastric tissues and explore the network of microbial interactions and influencing factors in GC tissue. The results would be helpful for future exploration of the relationship and mechanisms between microbiota and gastric cancer.

Previously, gastric tissue was universally considered to be devoid of microorganisms due to its prolonged exposure in a strong acidic environment^30^. However, during the development of sequencing technology, more evidences have been obtained to confirm that the stomach contains a diverse microbial community. A total of 1324 genera were detected across the four datasets in this study. The pan-microbiome was estimated to be open, the size of which might surpass 1400. Then we identified 17 core genera across the datasets, which were prevalent in gastric tissue. Among them, *Acinetobacter, Helicobacter, Pasteurella, Ralstonia, Bacillus*, *Pseudomonas*, *Clostridium* and *Cutibacterium* were also detected in gastric tissue through 16S rRNA sequencing or Meta-analysis in other studies^31–34^. In addition, other core microbiota had also been reported to be involved in tumorigenesis or tumor treatment. For example, Chiu’s study has demonstrated the anti-tumor activity capacity and inhibition of the growth of cancer cells of the secondary metabolites of *Streptomyces* sp.^35^. *Chlamydia* sp. has been confirmed to be involved in cell proliferation process and inhibiting apoptosis^36,37^. *Mycolicibacterium* sp., growing rapidly in vitro, has shown outstanding anti-tumor and immunomodulatory capabilities^38^. *Cutibacterium* sp. can induce long-term chronic infections in various areas including the prostate and has been identified as the dominant microbe in prostatic tissue obtained from prostate cancer patients^39^. From this perspective, we should pay more attention to the distribution of core or epidemic microbial groups in GC tissues, which might play a potentially important role in GC tumors.

Previous studies have shown that advanced age, male and environmental factors, such as smoking and eating smoked foods, all can increase the risk of gastric cancer^40^. However, we found that age and gender had no significant effect on the microbial composition of gastric cancer tissues, suggesting that the tumor microbiome might be in a stable state after tumorigenesis. In addition, we identified several microorganisms with significant differences between normal and tumor tissues. Among them, *Helicobacter pylori*, has been confirmed to increase the risk of gastric cancer and its abundance is significantly reduced in tumor tissues^10^. *Gemella*, *Pseudomonas, Acidovorax* are also common microorganisms enriched in gastric tumor tissues^33,41^. These are consistent with our results. Moreover, *Acidovorax* has been developed as a sputum biomarker to diagnose lung squamous cell carcinoma^42^. *Pseudomonas aeruginosa* was relatively abundant in the genus *Pseudomonas* and has been proved to be an important cause of infection in immunosuppressed patients, particularly cancer patients^43^. Dysregulation of these bacteria (*Gemella*, *Pseudomonas, Acidovorax*) may alter gastric cancer risk.

Notably, a series of differential genera related to tumor stage were identified. Among them, *Lysobacter* and *Kitasatospora* were significantly enriched in normal tissue and showed a significant increase trend in tumor development. A new proteasome inhibitor, derived from tyropeptin A produced by *Kitasatospora* sp., showed a strong growth inhibition and apoptosis in human prostate cancer^44^. Interestingly, we found *Acinetobacter*, *Pasteurella*, *Streptomyces* had higher relative abundance and showed a significant increase trend in the development of tumors, and formed strong correlations among them or with other genera. *Acinetobacter baumannii* detected in the genus *Acinetobacter* has been shown to be an opportunistic pathogen and carbapenem-resistant *Acinetobacter baumannii* infection in cancer patients has been shown associated with high mortality^45^. *Pasteurella multocida*, the most abundant bacterium detected in the genus *Pasteurella*, has been shown that the protein toxin produced by *Pasteurella multocida* is a potential carcinogen^46^. The *Streptomyces* sp. is the rich source of terpenoids, some of which have anti-tumor activity in against cancer cells^35^. In summary, these results suggest that changes in these gastric microbes (*Lysobacter*, *Kitasatospora*, *Acinetobacter*, *Pasteurella*, *Streptomyces*) may contribute to GC initiation and progression, so follow-up studies should focus on exploring the molecular mechanisms of these microorganisms on the occurrence and development of tumor.

However, our study has limitations. First, there are many other factors that may affect the gastric microbiota, such as dietary habits and previous diseases. The lack of biological information about these factors make it impossible to effectively explore their impacts. Second, the sample has strong individual heterogeneity and the sample size for analysis is not enough.

## Conclusion

To date, however, the composition of microbiota in gastric tissues is not clear and the gastric microbiome changes throughout the different stages of gastric carcinogenesis remain mostly elusive. This study revealed the microbiome of gastric cancer tissue and explored the influences of biological factors on gastric microecology. We find the microbial composition of the GC microenvironment is highly conservative and stable. Seventeen common core genera were identified in GC tissue, which should be paid more attention. Moreover, tissue type and tumor stage may play a significant role in the alteration of microbiome composition. Notably, we found significantly different genera in the occurrence and development of tumor. These bacteria (*Helicobacter*, *Streptomonospora*, *Acinetobacter*, *Pasteurella*, *Streptomyces*, *Chlamydia*, and *Lysobacter*) may be involved in tumor progression as potential characteristic genera and may provide a theoretical foundation for the non-invasive prediction of gastric cancer. More work is needed in the future to verify these bacteria in actual samples and explore the molecular mechanisms in tumorigenesis of gastric cancer.

## Supplementary Information

Supplementary Figure S1.

Supplementary Figure S2.

Supplementary Figure S3.

Supplementary Figure S4.

Supplementary Figure S5.

Supplementary Figure S6.

## Data Availability

The datasets analysed during the current study are available in the Gene Expression Omnibus (GEO), provided by Hwang D (SRP172499, RNA-seq, https://www.ncbi.nlm.nih.gov/geo/query/acc.cgi?acc=GSE122401), Xue Y (SRP337610, RNA-seq, https://www.ncbi.nlm.nih.gov/geo/query/acc.cgi?acc=GSE184336), Xue Y (SRP326473, RNA-seq, https://www.ncbi.nlm.nih.gov/geo/query/acc.cgi?acc=GSE179252) and Shen Z (SRP330001, RNA-seq, https://www.ncbi.nlm.nih.gov/geo/query/acc.cgi?acc=GSE180887).
