# Supplementary figures and images for: Uncovering the special microbiota associated with occurrence and progression of gastric cancer by using RNA-sequencing
Source: Sci Rep. 2023 Apr 7;13:5722. doi: 10.1038/s41598-023-32809-9 (PMC10082026; doi:10.1038/s41598-023-32809-9)

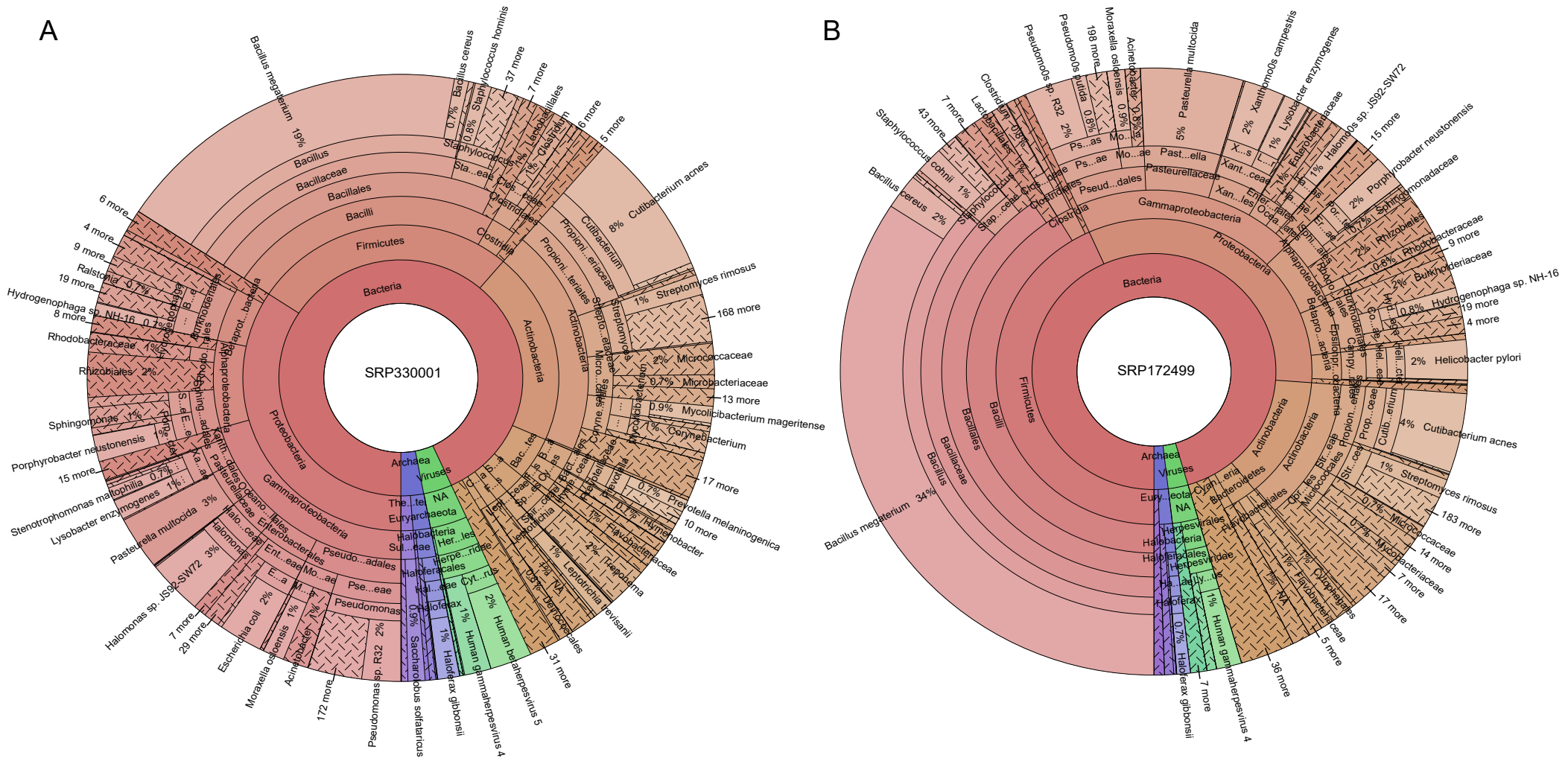

Supplement: Supplementary file 1 — Supplementary Figure S1. [file 41598_2023_32809_MOESM1_ESM.pdf]
