# Supplementary material for: Uncovering the special microbiota associated with occurrence and progression of gastric cancer by using RNA-sequencing
Source: Sci Rep. 2023 Apr 7;13:5722. doi: 10.1038/s41598-023-32809-9 (PMC10082026; doi:10.1038/s41598-023-32809-9)
Supplement: Supplementary file 2 — Supplementary Figure S2. [file 41598_2023_32809_MOESM2_ESM.pdf]

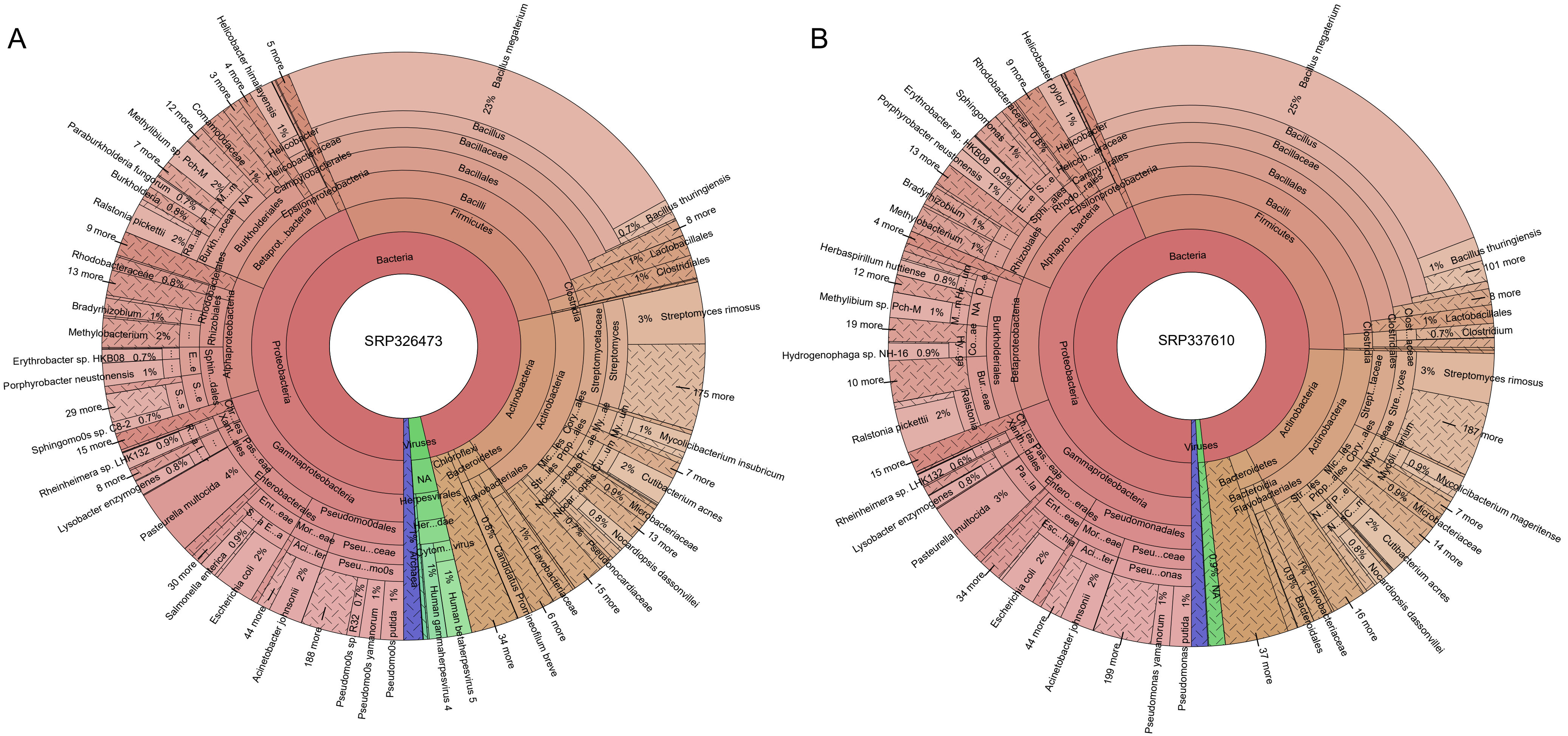

Supplemental Fig. 2 Krona multilayered pie-charts representing the taxonomic hierarchies of the microbial communities of the dataset SRP326473 (A) and SRP337610 (B).
