# Supplementary material for: Uncovering the special microbiota associated with occurrence and progression of gastric cancer by using RNA-sequencing
Source: Sci Rep. 2023 Apr 7;13:5722. doi: 10.1038/s41598-023-32809-9 (PMC10082026; doi:10.1038/s41598-023-32809-9)
Supplement: Supplementary file 3 — Supplementary Figure S3. [file 41598_2023_32809_MOESM3_ESM.pdf]

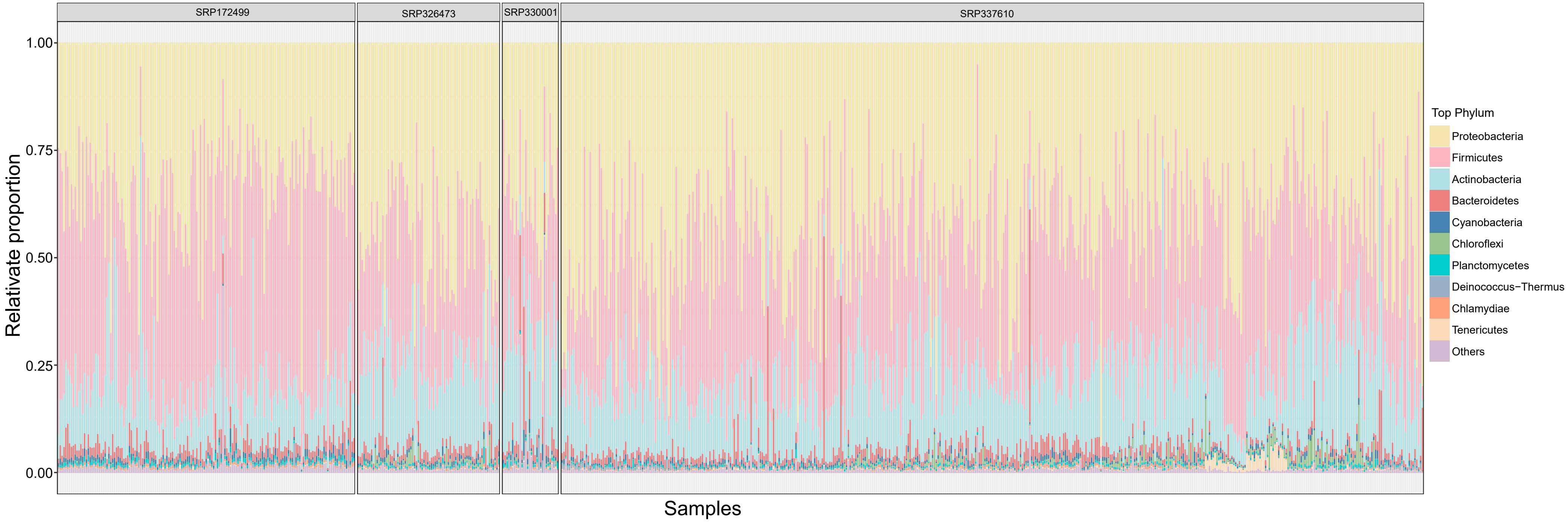

Supplemental Fig. 3 The relative abundances of top 10 phylum in all samples across the datasets. “Others” represents a collection of the taxa with lower relative abundances.
