# Supplementary material for: Uncovering the special microbiota associated with occurrence and progression of gastric cancer by using RNA-sequencing
Source: Sci Rep. 2023 Apr 7;13:5722. doi: 10.1038/s41598-023-32809-9 (PMC10082026; doi:10.1038/s41598-023-32809-9)
Supplement: Supplementary file 4 — Supplementary Figure S4. [file 41598_2023_32809_MOESM4_ESM.pdf]

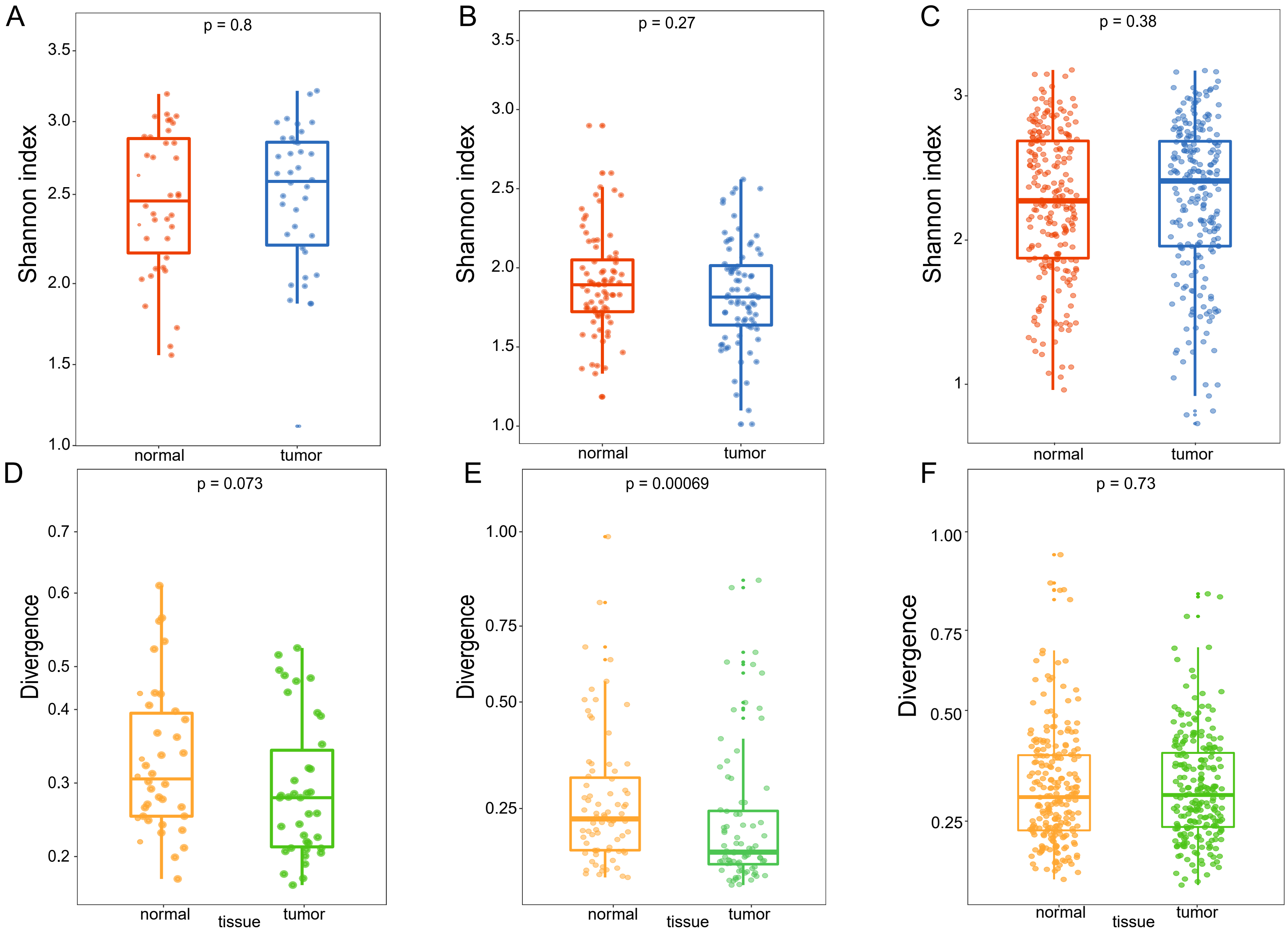

Supplemental Fig. 4 The distribution of Shannon index and the divergence distribution within the tissue type. The Wilcoxon signed rank test is used for the statistical differences. The distribution of Shannon index within the tissue type in the dataset SRP326473 (A), in the dataset SRP172499 (B) and in the dataset SRP337610 (C). The divergence distribution within the tissue type in the dataset SRP326473 (D), in the dataset SRP172499 (E) and in the dataset SRP337610 (F).
