# Supplementary material for: Uncovering the special microbiota associated with occurrence and progression of gastric cancer by using RNA-sequencing
Source: Sci Rep. 2023 Apr 7;13:5722. doi: 10.1038/s41598-023-32809-9 (PMC10082026; doi:10.1038/s41598-023-32809-9)
Supplement: Supplementary file 5 — Supplementary Figure S5. [file 41598_2023_32809_MOESM5_ESM.pdf]

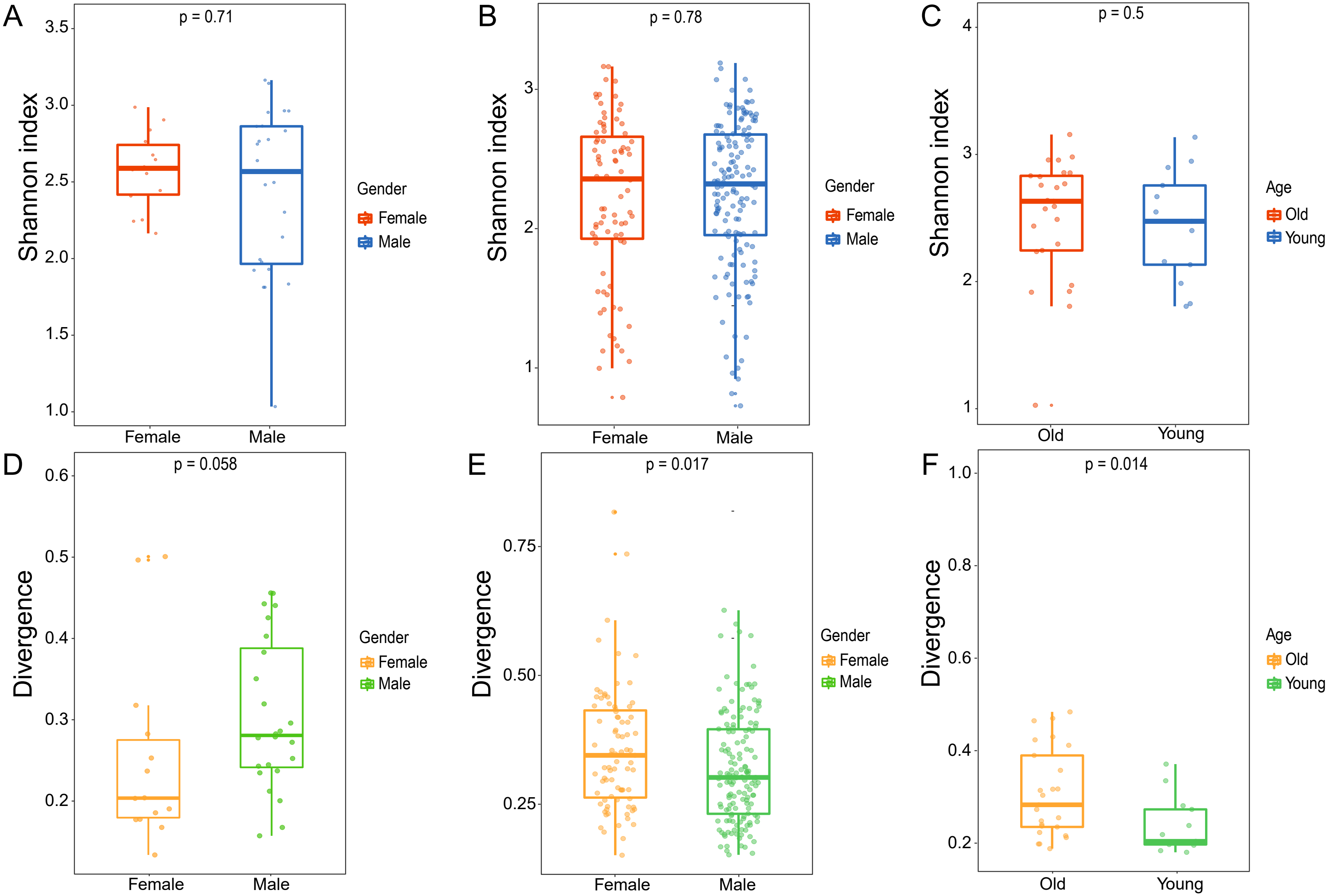

Supplemental Fig. 5 The distribution of Shannon index and the divergence distribution within the gender groups and age groups. The Wilcoxon signed rank test is used for the statistical differences. The distribution of Shannon index within the gender groups in the dataset SRP326473 (A) and in the dataset SRP337610 (B). The distribution of Shannon index within the age groups in the dataset SRP326473 (C). The divergence distribution within the gender groups in the dataset SRP326473 (D) and in the dataset SRP337610 (E). The divergence distribution within the age groups in the dataset SRP326473 (F).
