# Supplementary material for: Uncovering the special microbiota associated with occurrence and progression of gastric cancer by using RNA-sequencing
Source: Sci Rep. 2023 Apr 7;13:5722. doi: 10.1038/s41598-023-32809-9 (PMC10082026; doi:10.1038/s41598-023-32809-9)
Supplement: Supplementary file 6 — Supplementary Figure S6. [file 41598_2023_32809_MOESM6_ESM.pdf]

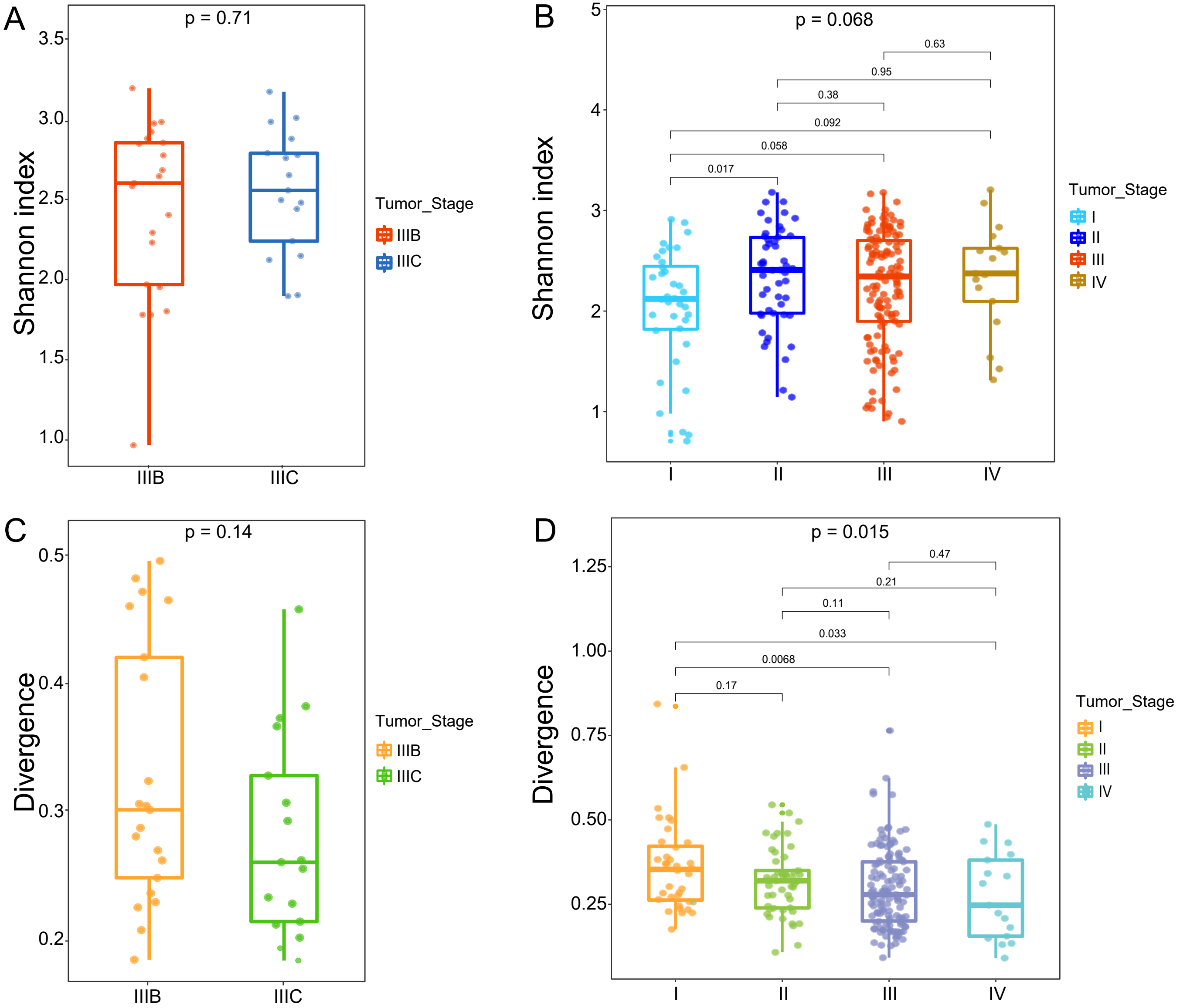

Supplemental Fig. 6 The distribution of Shannon index and the divergence distribution within the tumor stage. The distribution of Shannon index within the tumor stage in the dataset SRP326473 (A) and in the dataset SRP337610 (B). The divergence distribution within the tumor stage in the dataset SRP326473 (C) and in the dataset SRP337610 (D).
